# The long non-coding RNA TUG1 indicates a poor prognosis for colorectal cancer and promotes metastasis by affecting epithelial-mesenchymal transition

**DOI:** 10.1186/s12967-016-0786-z

**Published:** 2016-02-08

**Authors:** Junfeng Sun, Chaohui Ding, Zhen Yang, Tao Liu, Xiefu Zhang, Chunlin Zhao, Jiaxiang Wang

**Affiliations:** Gastrointestinal Surgery, The First Affiliated Hospital of Zhengzhou University, No.1 Jianshe East, Zhengzhou, 450052 China; Pediatric Surgery, The First Affiliated Hospital of Zhengzhou University, No.1 Jianshe East, Zhengzhou, 450052 China

**Keywords:** Colorectal cancer cell lines, EMT, HDACs, Metastasis, Taurine upregulated gene 1

## Abstract

**Background:**

Long intergenic non-coding RNAs (lncRNAs) are a class of non-coding RNAs that are involved in gene expression regulation. Taurine up-regulated gene 1 (TUG1) is a cancer progression related lncRNA in some tumor oncogenesis; however, its role in colorectal cancer (CRC) remains unclear. In this study, we determined the expression patterns of TUG1 in CRC patients and explored its effect on CRC cell metastasis using cultured representative CRC cell lines.

**Methods:**

The expression levels of TUG1 in 120 CRC patients and CRC cells were determined using quantitative real-time PCR. HDACs and epithelial-mesenchymal transition (EMT)-related gene expression were determined using western blot. CRC cell metastasis was assessed by colony formation, migration assay and invasion assay.

**Results:**

Our data showed that the levels of TUG1 were upregulated in both CRC cell lines and primary CRC clinical samples. TUG1 upregulation was closely correlated with the survival time of CRC patients. Overexpression of TUG1 in CRC cells increased their colony formation, migration, and invasion *invitro* and promoted their metastatic potential *in vivo*, whereas knockdown of TUG1 inhibited the colony formation, migration, and invasion of CRC cells *invitro*. It is also worth pointing out that TUG1 activated EMT-related gene expression.

**Conclusion:**

Our data suggest that tumor expression of lncRNA TUG1 plays a critical role in CRC metastasis. TUG1 may have potential roles as a biomarker and/or a therapeutic target in colorectal cancer.

## Background

Colorectal cancer (CRC) remains a primarily world-wide health concern in spite of significant improvements in its diagnosis and therapy modalities. Over 1.2 million new CRC cancer cases and 608,700 deaths are recorded annually [1]. Historically, comprehensive cancer treatment in cases of cancer metastases has always been very challenging [2]. Currently, the need to elaborate knowledge on the underlying molecular mechanisms for cancer metastasis in CRC is urgently needed.

Mammalian genomes encode a wide variety of conserved non-coding RNA transcripts [3, 4]. In addition to classical ‘housekeeping’ RNAs (such as ribosome RNAs, transfer RNAs, and others) and widely-defined microRNAs, long non-coding RNAs (lncRNAs) have recently been identified as one of fraction of untranslated RNA molecules. lncRNAs, transcribed by RNA polymerase II (RNA pol II), are characterized by lengths of 200 nucleotides to ~100 kilobases (kb) and by their lack of a significant open reading frame [3]. These mRNA-like molecules are pervasively transcribed and roughly classified as antisense, based on their position relative to the protein-coding genes [5] and exhibit cis- or trans- regulatory capabilities for gene expression. Gene expression patterns indicate that these lncRNAs are implicated in diverse biological processes, including nuclear architecture, regulation of gene expression, immune surveillance, or embryonic stem cell pluripotency. Recently, evidence revealing the molecular mechanisms by which these RNA species function has provided some insight into the functional roles they may play in tumorigenesis [6]. Aberrant lncRNA expression participates in carcinogenesis by disrupting major biological processes, such as redirecting chromatin remodeling complexes or inactivating major tumor suppressor genes [6, 7].

Among these, LincRNA taurine up-regulated gene 1 (TUG1; also known as TI-227H; Linc00080; ncRNA00080) was originally identified as a transcript up-regulated by taurine and is found to be expressed in various human cancer cell lines and tumors [8]. Upregulation of the long noncoding RNA TUG1 promotes proliferation and migration of esophageal squamous cell carcinoma [9]. Downregulation of long non-coding RNA TUG1 inhibits osteosarcoma cell proliferation and promotes apoptosis [10]. However, the role of TUG1 in colorectal cancer remains unclear. This study was established due to our speculation that TUG1 may have roles to play in the CRC pathological process.

Previously, research has found that the metastasis and invasion of cancer cells are common events that mediate changes in cellular behavior, and induce different steps in the metastatic cascade [11, 12]. One of the most crucial steps in the tumor cell metastatic cascade is the acquisition of invasive capabilities, including destroying cell–cell junctions, degrading the cell matrix, and activating pathways that control the cytoskeletal dynamics of cancer cells. Over the past decade, cell and tumor biologists have identified the key role of epithelial-mesenchymal transition (EMT)-a biological process in which epithelial cells lose their polarity and transition into a mesenchymal phenotype-in cancer cell metastasis [13]. Evidence suggests that EMT enhances tumor cell invasion in response to environmental triggers, augments invasive functions, and also contributes to cell growth and survival [14, 15].

In preliminary experiments, we found that upregulated UTR1 expression in metastatic CRC cell lines predicted poor survival for CRC patients. This current study was designed to search the evidence supporting a role for TUG1 in the metastasis of CRC in representative CRC cell lines. We further attempted the preliminary exploration of the possible association of EMT-related gene expression by TUG1.

## Methods

### Clinical samples

We obtained paired CRC tumor samples (bulk samples) and adjacent non-tumor colorectal tissues from 120 patients who had undergone gastrointestinal surgery between 2008 and 2013 at the First Affiliated Hospital of Zhengzhou University, China. All specimens were immediately frozen in liquid nitrogen and stored at 80 °C until total RNA extraction. Written informed consent was obtained from all patients. No patient received chemotherapy or radiotherapy before surgery. The follow-up periods ranged from 2 months to 5 years, with a mean of 3 years. Our study was approved by the Research Ethics Committee of Zhengzhou University.

### Cell lines and cultivation

Human colorectal cancer cell lines including SW480, HCT116, HT29, SW620, and LOVO, were obtained from the Key Laboratory of Cancer Prevention and Intervention, Cancer Institute, The Affiliated Hospital, Zhengzhou University School of Medicine, Zhengzhou, China. The SW480, SW620, and LOVO cell lines were cultured in L-15 (with 10 % FBS and 1 % streptomycin/penicillin); HCT-116 cell lines were cultured in McCoy’s 5A (with 10 % FBS and 1 % streptomycin/penicillin); and HT-29 cell lines were cultured in RPMI-1640 (with 10 % FBS and 1 % streptomycin/penicillin). Normal human colorectal cells were purchased from the American Type Culture Collection and cultured in RPMI1640 supplemented with 10 % FBS and 2 mM l-Glutamine (Gibco). All cell lines were maintained at 37 °C and 5 % CO_2_ in an incubator, and passaged with 0.25 % trypsin (Sigma, St Louis, MO, USA) in 0.2 mol/l phosphate-buffered saline (PBS; Sigma). The study was approved by the ethics committee of the Cancer Institute, The Affiliated Hospital, Zhengzhou University School of Medicine, Zhengzhou, China.

### Real-time quantitative PCR

Total RNA was extracted from colorectal tumor samples and CRC cell lines using TRIzol reagent (Invitrogen, USA) according to the manufacturer’s protocol. Complementary DNA was synthesized from total RNA with the Revert Aid™ First Strand cDNA Synthesis Kit (Thermo Scientific, USA). The primer sequences were as follows: TUG1 forward primer: 5c-TAGCAGTTCCCCAATCCTTG-3′, reverse primer: 5′-CACAAATTCCCATCATTCCC-3′; GAPDH forward primer: 5′-CGCTCTCTGCTCCTCCTGTTC-3′, GAPDH reverse primer: 5′-ATCCGTTGACTCCGACC-TTCAC-3′. The PCR was performed in a total reaction volume of 20 ml and was completed in the ABI PRISM 7000 Fluorescent Quantitative PCR System (Applied Biosystems, Foster City, CA, USA). GAPDH was used as an internal control. The PCR cycling parameters were: one denaturation step of 10 min at 95 °C; 40 cycles, with one cycle consisting of 15 s at 95 °C, 20 s at 55 °C, and 30 s at 70 °C. The median in each triplicate was used to calculate the relative TUG1 expression level using the comparative DCt method (value of 2^−DCt(TUG1-GAPDH)^). Expression fold changes were calculated using 2^−DDCt^ methods.

### Protein isolation and western blotting

For the protein expression analyses, standard western blot assay was carried out. Cultured or transfected cells were washed twice with cold phosphate-buffered saline (PBS) and were lysed with iced RIPA buffer containing 1 % PMSF (KeyGen, Nanjin, China). After total protein detection using a BCA kit (Beyotime, Shanghai, China), protein lysates were separated on 10 % SDS polyacrylamide gel, transferred to PVDF membranes, and blocked in 0.1 % Tween 20 and 5 % skim milk protein in Tris Buffer Saline at room temperature for 2 h. Target proteins were probed with rabbit anti-HDAC1 antibody (1:800; Proteintech Corporation, USA), rabbit anti-HDAC2 antibody and rabbit anti-HDAC3 monoclonal antibody (1:1000; Bioworld Corporation, USA), and mouse anti-E-cadherin, N-cadherin, fibronectin, vimentin (1:1000; Abcam, USA), and rabbit anti-β-Actin antibody (1:2000; acted as an internal control, Zhongshan, Inc., Beijing, China) overnight at 4 °C. The membranes were washed twice and visualized with horseradish peroxidase (HRP)-conjugated secondary antibodies for 2 h. Tagged proteins were detected by enhanced chemiluminescence (KeyGen, Nanjing, China).

### Plasmid and lentivirus vector

Ectopic expression of TUG1 in cells was realized through pcDNA-TUG1 transfection. The TUG1 sequence was synthesized and subcloned into the pCDNA3.1 (Invitrogen, Shanghai, China) vector. The empty pcDNA3.1 vector was used as a control. To assess the effect of TUG1 on liver metastasis *invivo*, TUG1 was overexpressed by SW480 transfecting with Lenti-GIII-CMV-Human-TUG1 Lentivirus [Applied Biological Materials (ABM) Inc, Canada]. Following this step, the expression levels of TUG1 were detected by quantity real-time PCR.

### Cell transfection

Plasmid vector (pcDNA-TUG1 and pCDNA3.1) or lentivirus vector (Lenti-GIII-CMV-TUG1 and Lenti-GIII-CMV) were prepared using DNA Midiprep or Midiprep kits (Qiagen, Hilden, Germany), and were transfected into SW480 cells. Two individual siRNAs (TUD1-siRNA and HDAC1-siRNA) and negative control siRNA (silencer negative control siRNA) were purchased from Ambion. siRNA oligonucleotides (10 nmol/L) in Opti-MEM (Invitrogen) were transfected into LOVO cells or SW480 cells, respectively, using Lipofectamine RNAiMAX (Invitrogen) and following the manufacturer’s protocol. Forty-eight hours post-transfection, TUD1 and HDAC1 expression levels were measured, and/or cell metastasis ability was assessed. Target sequences for siRNAs were as follows: TUG1-siRNA1 (sense 5′-GGGAUAUAGCCAGAGAACAAUUCU-A-3′, antisense 5′-UAGAAUUGUUCUCUGGCUAUAUCCC-3′) and HDAC1-siRNA, sense 5′-CCAAGTACCACAGCGATGAC-3′, and antisense 5′-TGGACAGTCCTCACCAACG-3′.

### Plate clone formation assay

Each well in a 6-well culture plate was seeded with 10^2^ cells and each group contained three wells. The cells were incubated at 37 °C with 5 % CO_2_ for 2–3 weeks and the medium was replaced every 3 days. The incubation was stopped when colony formation was visible and the cells were then washed twice with PBS and stained with Giemsa solution for 20 min. The number of colonies containing 50 cells was counted under a microscope using the formula: plate clone formation efficiency = (number of colonies/number of cells inoculated) × 100 %.

### Wound healing assay

SW480^pcDNA-TUG1^, LOVO^si-TUG1^ and their corresponding control cells were separately seeded into 6-well plates, and were cultured until they were nearly 80 % confluent. Artificial wounds were created by scraping the monolayers with a sterile 10 ml tip, and the cells were washed with PBS several times to remove floating cells. Representative images of cells migrating into the wounds were captured after 0 and 24 h under a microscope (200×).

### Cell migration assays

Cell migration assays were performed using 24-well Transwell plates (8.0-μm pore membranes; Costar). About 1 × 10^4^ cells (SW480^pcDNA-TUG1^ or LOVO^si-TUG1^) were loaded into the upper chambers. The lower chambers were filled with a medium (plus 1 % FBS) in the absence (DMSO 0.2 %) or presence of SecinH3 (10, 20, or 40 mM). The Transwell plates were then incubated in a 37 °C, 5 % CO_2_ incubator for 48 h. After cleaning the cells from the upper side of the polycarbonate membrane and following hematoxylin–eosin staining, the polycarbonate membrane was cut and placed on a microscope slide, cover slipped, and examined under the microscope. The migrated cell numbers and percentages were then counted.

### Matrigel invasion assay

The Matrigel invasion assay was done using the BD Biocoat Matrigel Invasion Chamber (pore size: 8 mm, 24-well; BD Biosciences, San Jose, CA, USA) following the manufacturer’s protocol. Cells (5 × 10^4^) were plated in the upper chamber in a serum-free medium. The bottom chamber contained a medium with 10 % FBS. After the 48 h incubation, the bottom of the chamber insert was stained with Calcein AM (Invitrogen). The cells that had invaded through the membrane to the lower surface were evaluated in a fluorescence plate reader at excitation/emission.

### Experimental *invivo* liver metastasis model

Female athymic BALB/c nude mice (aged 6 weeks) were purchased from the Shanghai Laboratory Animal Center Co. Ltd. (Shanghai, China) and maintained in a pathogen-free animal facility at the Laboratory Animal Research Centre of Zhengzhou University. For liver metastatic capacity, the spleen of BALB/c nude mice was injected with 1 × 10^6^ cells per mouse. Briefly, BALB/c nude mice were anesthetized by i.p. injection of Pelltobarbitalum Natricum, and 1 × 10^6^ SW480^pcDNA3.1^ or SW480^pcDNA-TUG1^ tumor cells in 25 ml were injected into the exteriorized spleen using an insulin syringe and following abdominal incision. Five minutes after injection, spleen blood vessels were ligated, and the spleen was removed. Finally, the abdominal wound was closed with staples. After 5 weeks, mice were sacrificed and their livers were removed and tumor nodules were numbered.

### Statistical analysis

Statistical analysis was performed using GraphPad Prism 5.01 software. Statistical tests for data analysis included the log-rank test, the Chi square test. Multivariate statistical analysis was performed using a Cox regression model. The quantitative data were presented as the mean ± standard deviations (SD). Differences were considered to be statistically significant at values of *P* < 0.05.

## Results

### Upregulation of TUG1 is correlated with CRC progression

**Fig. 1:**
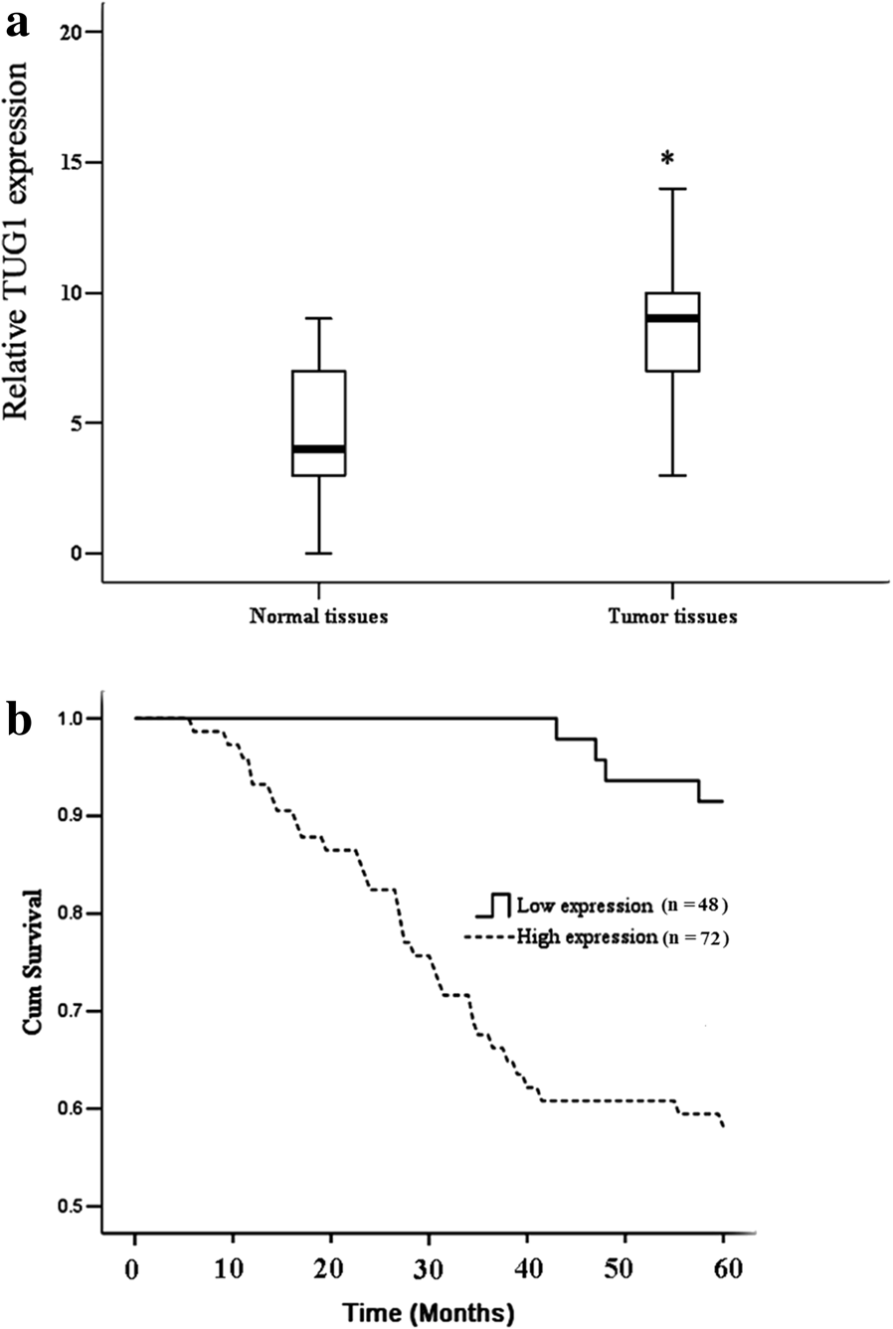
TUG1 is upregulated in primary human CRC samples. **a** Statistical analysis was performed to present TUG1 expression in paired para-tumor/tumor samples in each clinical sample test. **b** Kaplan–Meier curves of CRC patients with low versus high expression of TUG1 (n = 120, *P* < 0.001, log-rank test); high expression showed that tumor TUG1 expression is 5 times higher than para-tumor tissue; low expression showed that tumor TUG1 expression is 5 times lower than para-tumor tissue. Values represent mean ± SD. * *P* < 0.05 compared with normal tissues

To investigate the clinical relevance of abnormal TUG1 expression in CRC, we collected 120 pre-frozen primary CRC tissue samples (Table 1), and determined TUG1 expression levels in all of these samples using quantitative real-time PCR. By data statistics and analysis, we observed that TUG1 expression was significantly elevated in tumor tissues compared with para-tumor tissue (Fig. 1a), and univariate analysis revealed a close association between high TUG1 expression and patient survival (Fig. 1b). These data suggest that TUG1 upregulation is strongly associated with the clinical progression of human CRC.

**Table 1:** TUG1 (taurine upregulated gene 1) expression and clinicopathologic factors (n = 120)

Characteristics	All cases	TUG1 expression levels		
		Tumor high expression	Tumor low expression	P value
Age				0.512
<60	61	38	23	
≥60	59	33	26	
Gender				0.761
Male	80	60	20	
Female	40	29	11	
Grade				0.027*
Well and moderately	86	14	72	
Poorly differentiated	34	20	14	
Depth of tumor				0.034*
m, sm, mp	30	27	3	
ss, se, si	90	65	25	
Lymph node-metastasis				0.013*
Negative	70	23	47	
Positive	50	35	15	
Liver metastasis				
Negative	95	25	70	0.01*
Positive	25	18	7	
Dukes’ stage				
A and B	69	50	19	0.234
C and D	51	36	15	

Tumor high expression, the relative expression level of TUG1 is fivefold than para-carcinoma tissue. Tumor low expression, the relative expression level of TUG1 is less than fivefold than para-carcinoma tissue

*Difference is statistically significant

### Possible negative regulating TUG1 expression by histone deacetylase

**Fig. 2:**
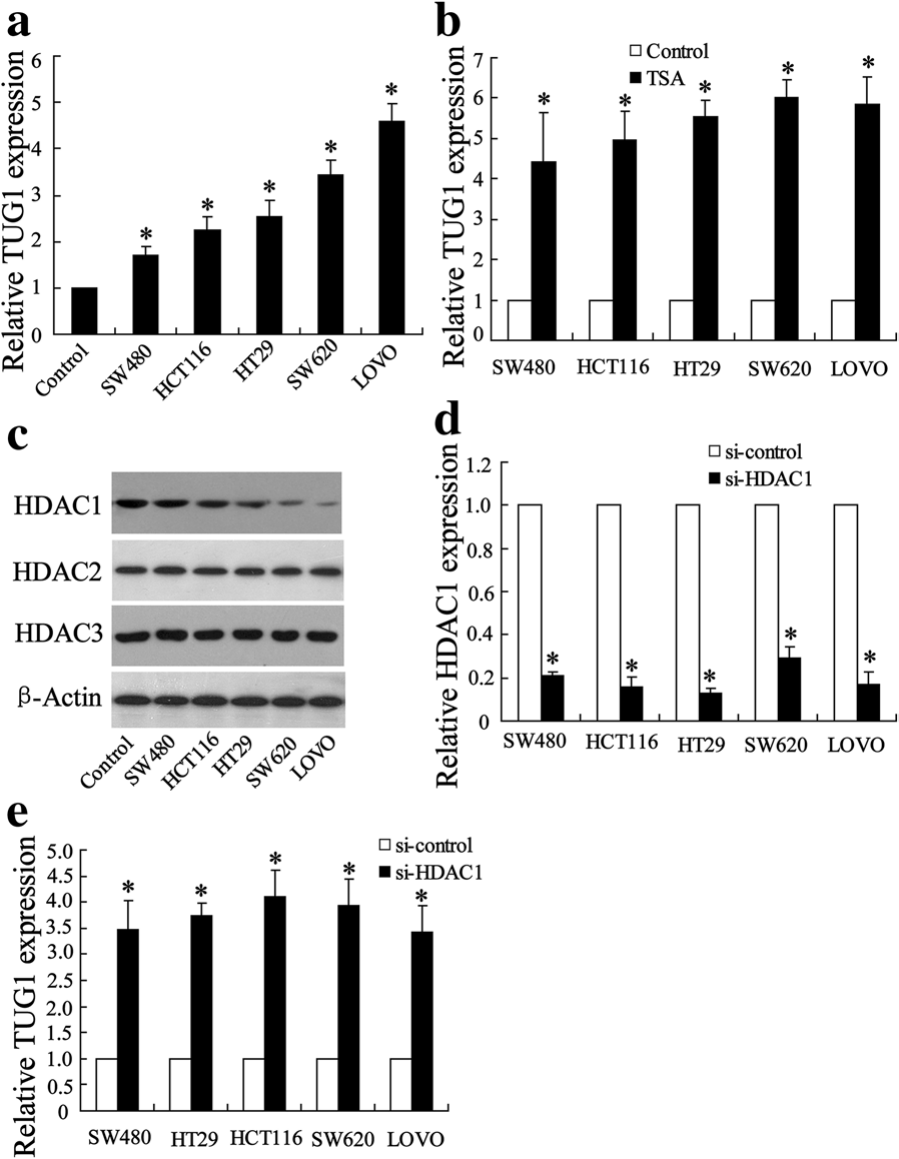
Enhanced expression of TUG1 by histone deacetylase inhibition. **a** Relative ectopic expression of TUG1 in normal colorectal cell (control) and colorectal cancer cell lines by using Q-RT-PCR. **b** Relative expression of TUG1 in TSA treated normal tissue (control) and colorectal cancer cell lines. **c** Western blot analysis of HDAC1, HDAC2, and HDAC3 expression in normal colorectal cell (control) and colorectal cancer cell lines; β-actin served as a loading control. **d** Knock down of HDAC1 by si-HDAC transfection in normal colorectal cells (control) and colorectal cancer cell lines. **e** Relative expression of TUG1 in si-HDAC treated normal colorectal cells (control) and colorectal cancer cell lines. The colorectal cancer cell lines containing SW480, HCT116, HT29, SW620, and LOVO. Values represent mean ± SD. * *P* < 0.05 compared with control or si-control

Next, we attempted to investigate the possible mechanism underlying aberrant TUG1 expression in CRC. The five representative CRC lines of SW480, HCT116, HT29, SW620, and LOVO were used for following analysis. We observed that TUG1 expression was significantly elevated in all CRC lines compared to normal colorectal cells (Fig. 2a). Interestingly, TUG1 expression was found to be 4–6 times higher than in corresponding cell lines (marked as control with no TSA treatment in presence of TSA, an inhibitor for histone deacetylase (Fig. 2b). We then evaluated HDAC1, HDAC2, and HDAC3 expression in these CRC lines in comparison to control and we found that HDAC1 expression was upregulated, while the expression levels of HDAC2 and HDAC3 protein remained unchanged in CRC cells (Fig. 2c). These data suggested that TUG1 expression was possibly regulated by abnormal expression HDAC1 expression. Thus, the CRC lines were used to create sub-lines with HDAC1 stably knocked down by shRNA focusing on analysis of TUG1 expression. From the result, we observed that HDAC1 silencing induced promotion (3.4–4.0 times the level of the si-control) of TUG1 expression (Fig. 2d).

### TUG1 promotes the aggressiveness of CRC cells *in vitro*

**Fig. 3:**
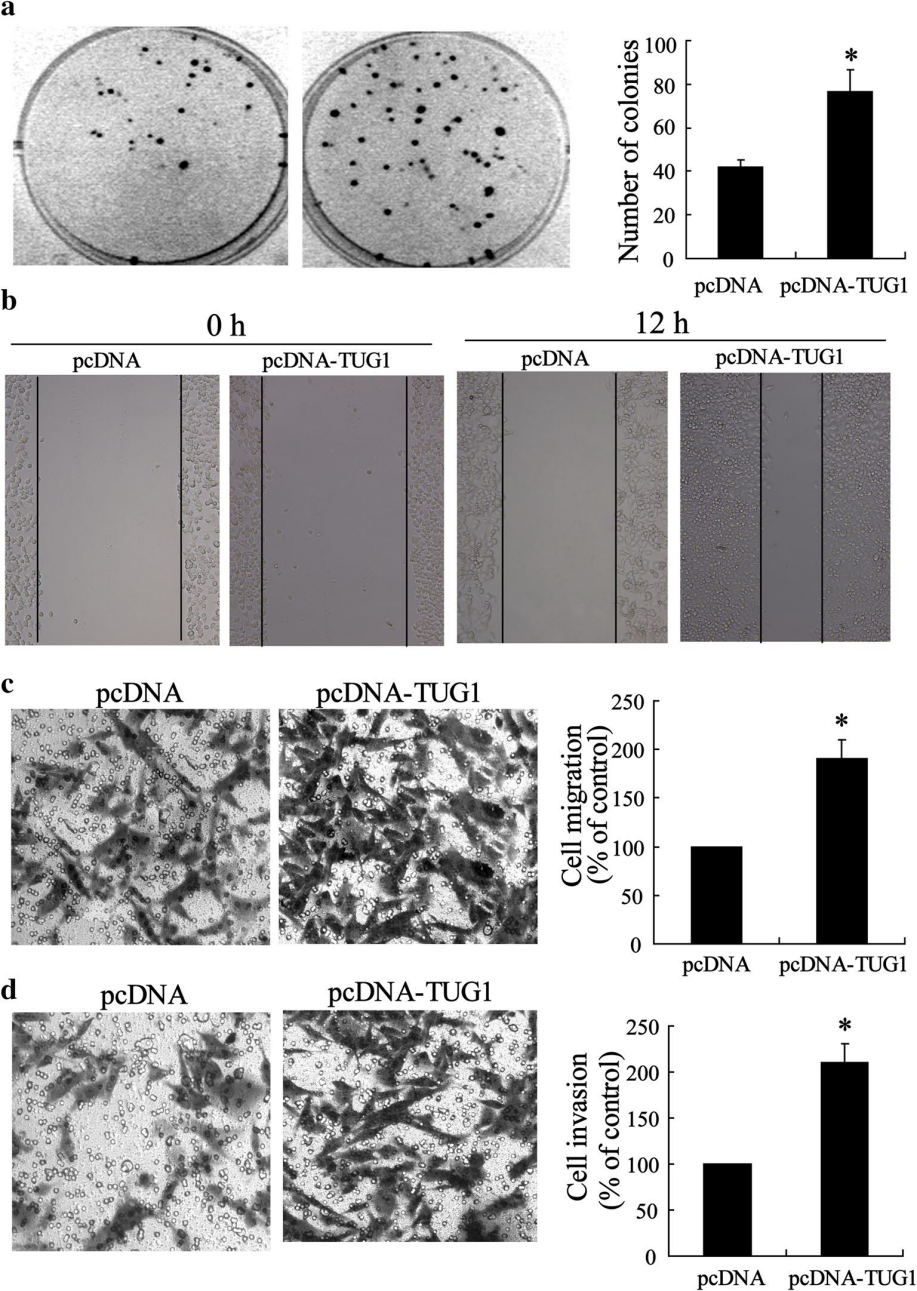
Enhanced metastasis of CRC cells with overexpressed TUG1. **a** Representative image and number statistics for colony formation in SW480^pcDNA^ and SW480^pcDNA-TUG1^ cells. **b** Wound-healing assay for motility of SW480^pcDNA^ and SW480^pcDNA-TUG1^ cells. Representative pictures of one field at the beginning (t = 0) (*left panel*) and at the end of the recording (t = 12 h) (*right panel*) in each condition. **c** Representative images of transwell migrated cells and **d** invaded cells in stably transfected SW480^pcDNA^ and SW480^pcDNA-TUG1^ cells and average number of migrated cells and invaded cells are shown in the *right* of (**c**) and (**d**). Values represent mean ± SD. * *P* < 0.05 compared with pcDNA

Considering that TUG1 has a strongly correlation with CRC pathological courses, we transfected the SW480 cell line with pcDNA-TUG1 and detected the effect of overexpressed TUG1 on CRC cell metastasis capacity. The results showed that TUG1 overexpression induced an increase in colony formation (Fig. 3a) and caused a significant facilitation of cell migration in a wound-healing assay (Fig. 3b). We also utilized the classical Transwell assay to assess the contribution of TUG1 to cell migration and invasion. As shown in Fig. 3c and d, overexpressed TUG1 increased migration of SW480 cells and enhanced cell invasion ability.

### Knock-down TUG1 suppresses CRC cell metastasis *invitro*

**Fig. 4:**
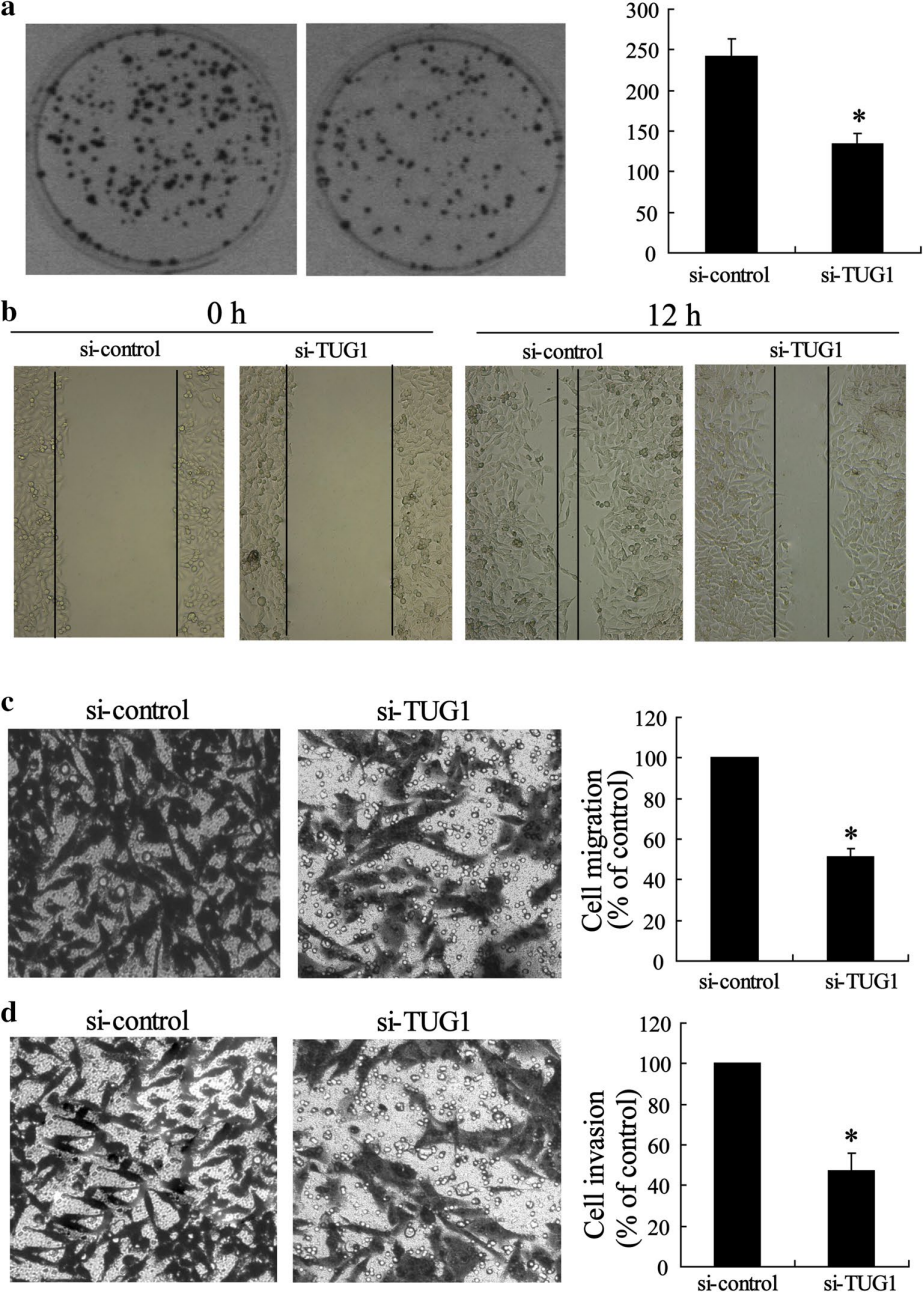
Silenced TUG1 inhibited metastasis of CRC cells. **a** Representative image and number statistics for colony formation in LOVO^si-control^ and LOVO^si-TUG1^ cells. **b** Wound-healing assay for motility of LOVO^si-control^ and LOVO^si-TUG1^ cells. Representative pictures of one field at the beginning (t = 0) (*left panel*) and at the end (t = 12 h) (*right panel*) of the recording in each condition are shown. **c** Representative images of transwell migrated cells, and **d** invaded cells in stably transfected LOVO^si-control^ and LOVO^si-TUG1^ cells and the average number of migrated cells and invaded cells are shown in the* right* of (**c**) and (**d**). Values represent mean ± SD. * *P* < 0.05 compared with si-control

Another CRC cell line- LOVO was used to establish cell subline with TUG1 stably knocked down by TUG1-shRNA transfection with the purpose of evaluating CRC cell metastasis ability. The results showed that, compared with the si-control, TUG1 silencing reduced the number of colony formations (Fig. 4a). TUG1 silencing also promotes cell migration showed by a wound-healing assay (Fig. 4a). Additionally, by classical Transwell assay, we observed that TUG1-shRNA interference caused significant suppression in ability of cell migration and invasion as shown in Fig. 4c and d.

### Inhibition of mice CRC progression by enhancing tumor-expressing TUG1

**Fig. 5:**
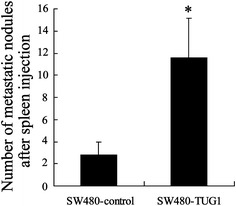
Statistics for mice metastatic nodules *in* *vivo*. Nude mice were injected with SW480^pcDNA^ or SW480^pcDNA-TUG1^ cells and tumor nodules were numbered 7 days post-transplantation. Values represent mean ± SD. * *P* < 0.05 compared with SW480^-^control

Liver metastasis of colon cancer is a common clinical phenomenon. Considering the identification of the key regulator role of TUG1 on CRC cell metastasis *invitro*, we next established an *invivo* experimental liver metastasis model by injecting human SW480 CRC cells into the spleens of BALB/c nude mice and followed their ability to invade-via the portal vein-the liver to form metastases and then investigated whether TUG1 plays an important role in the liver metastasis of CRC. To define the relationship between TUG1 and CRC liver metastasis *in vivo*, six, 7-week-old female BALB/c nude mice in each group were injected with SW480^pcDNA-TUG1^ or SW480^pcDNA^ cells into the spleen before splenectomy. After 5 weeks, the mice were sacrificed, and the metastatic tumor nodules that formed in the liver were numbered. The data showed that metastatic tumor nodules were more frequently found in the livers of the SW480^pcDNA-TUG1^ group than in the SW480^pcDNA^ group (Fig. 5).

### TUG1 facilitates epithelial-mesenchymal transition (EMT) in CRC cell lines

**Fig. 6:**
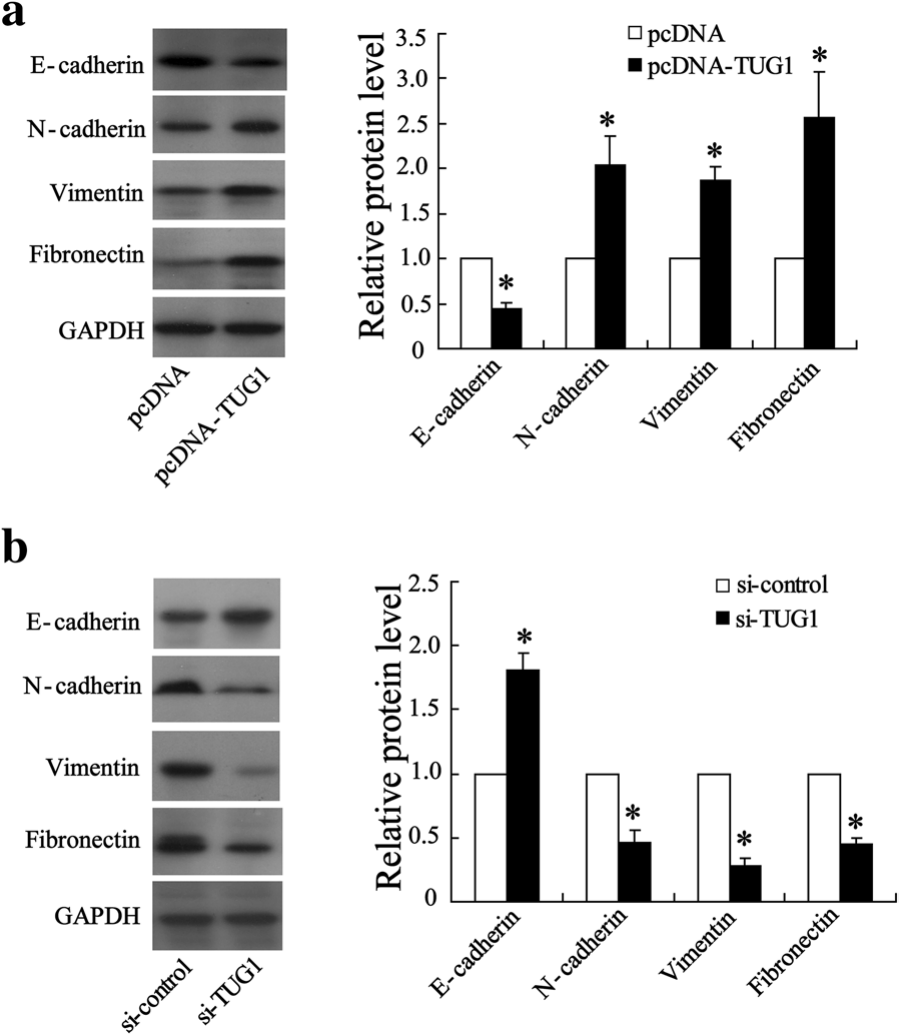
Effect of TUG1 on EMT related-gene expression in colorectal cancer cell line of SW480 and LOVO. **a** Western blot analysis of EMT-related gene expression in pcDNA-TUG1-transfected SW480 cells. **b** Western blot analysis of EMT-related gene expression in si-TUG1-transfected LOVO cells. Values represent mean ± SD. * *P* < 0.05 compared with pcDNA or si-control

EMT is an important factor in cell invasion. Thus, we next determined whether EMT markers were altered in our model. The expression of E-cadherin, N-cadherin, vimentin, and Fibronectin protein level was analyzed by Western blot. By data, we found that the expression of N-cadherin, vimentin and Fibronectin was increased while E-cadherin expression was decreased, in SW480-TUG1 cells (Fig. 6a), whereas the opposite results were obtained when TUG1 was knocked down in LOVO cells (Fig. 6b).

## Discussion

Recent evidence has shown that lncRNAs play an important role in cancer pathogenesis, and could provide new insights into the biology of this disease [7]. Although lncRNAs may have an impact on various human diseases, including cancer, the basis of their molecular mechanism is still beginning to be elucidated [16]. Likewise, no data are available for the role of them played during the CRC pathological process. One of these lncRNAs is metastasis-associated TUG1. Although TUG1 is recognized as the highly conserved nuclear lncRNA and is a predictive marker for metastasis development in human cancer [17], its role in CRC metastasis remains unclear.

In our current study, we analyzed the behavior of TUG1 in CRC metastasis. Initially, we observed that TUG1 expression levels in CRC tissues were higher than those in corresponding para-carcinoma tissues. As for clinicopathologic variables, TUG1 expression levels were speculated to be linked to colonic carcinogenesis. We thus chose five representative cell lines of CRC and investigated TUG1 levels with a non-tumoral colorectal cell line as control. By *invitro* data, we then showed that TUG1 overexpression significantly enhanced tumor-like characteristics by increasing the colony formation, migration, and invasion of CRC cells. Accordingly, the opposite results were obtained when TUG1 was knocked down. These results indicate that TUG1 might play a key role in promoting metastasis of CRC, which was further proven by a mice liver metastasis model in which TUG1 overexpression significantly increased the number of metastatic tumor nodules in the liver. Our study is consistent with previous research revealing that the high expression of TUG1 in primary CRC was strongly associated with lung metastases [17]. Moreover, our data showed that high TUG1 expression in CRC tissues was closely associated with a decreased survival time in CRC patients. These multivariate analyses suggested that TUG1 might be an independent risk factor for CRC metastasis.

Knowledge of how lncRNAs are regulated in complex gene regulatory systems has attracted a lot of attention. Previously, hypermethylation of the promoter or the intergenic differentially methylated region has been found to contribute to reduced expression of lncRNA MEG3 in tumors, indicating that epigenetic regulation is also involved in the expression of these genes [18]. The fact that whether histone deacetylation that functioned as epigenetic regulatory factors manipulate the expression of TGU1 remains unknown. Our findings emphasize that histone deacetylase is a key factor in controlling the expression of the lncRNA TUG1. We observed that both TSA (an inhibitor for histone deacetylase) and HDACs knockdown enhanced THG1 expression. These results, along with those from a recent study [19], highlight the role of epigenetics in regulating lncRNA transcription.

To explore the molecular mechanism through which TUG1 contributes to the invasion and metastasis of CRC cells, we investigated potential target proteins involved in cell motility and matrix invasion, such as EMT-related gene expression. EMT is essential for cancer cell metastasis and it enhances tumor cell invasion in response to environmental triggers, and augments invasive functions and also contributes to cell growth and survival [20, 21]. Important hallmarks of EMT include the loss of E-cadherin expression and increased expression of non-epithelial cadherins, such as N-cadherin and vimentin. The loss of E-cadherin expression is a fundamental event in EMT, and a crucial step in the progression of papillomas to invasive carcinomas [13]. Therefore, we determined the protein levels of these EMT-induced markers following ectopic expression of TUG1. Our results indicated that TUG1 overexpression reduced E-cadherin expression and enhanced the expression levels of N-cadherin, vimentin, and Fibronectin, whereas knockdown of endogenous TUG1 expression significantly abrogated these capacities. These data indicated that TUG1 might influence CRC metastasis by mediating EMT-related gene expression.

## Conclusion

In summary, the expression of TUG1 was significantly increased in CRC tumor tissues, suggesting that its downregulation may be a negative prognostic factor for CRC patients, and indicative of poor survival rates and a higher risk for cancer metastasis. We showed that TUG1 possibly regulates the invasive and metastatic ability of CRC cells, partially through regulation of EMT. Our findings promote further the understanding of CRC pathogenesis and development, and facilitate the development of lncRNA-directed diagnostics and therapeutics against cancers. However, the molecular mechanisms by which HDAC1 controls TUG1 expression and TUG1 regulates EMT require further investigation.

## Abbreviations

lncRNAs: long intergenic non-coding RNAs
TUG1: taurine up-regulated gene 1
CRC: colorectal cancer
EMT: epithelial-mesenchymal transition
RNA pol II: RNA polymerase II
kb: kilobases
PBS: phosphate-buffered saline
HRP: horseradish peroxidase
ABM: applied biological materials
SD: standard deviations
