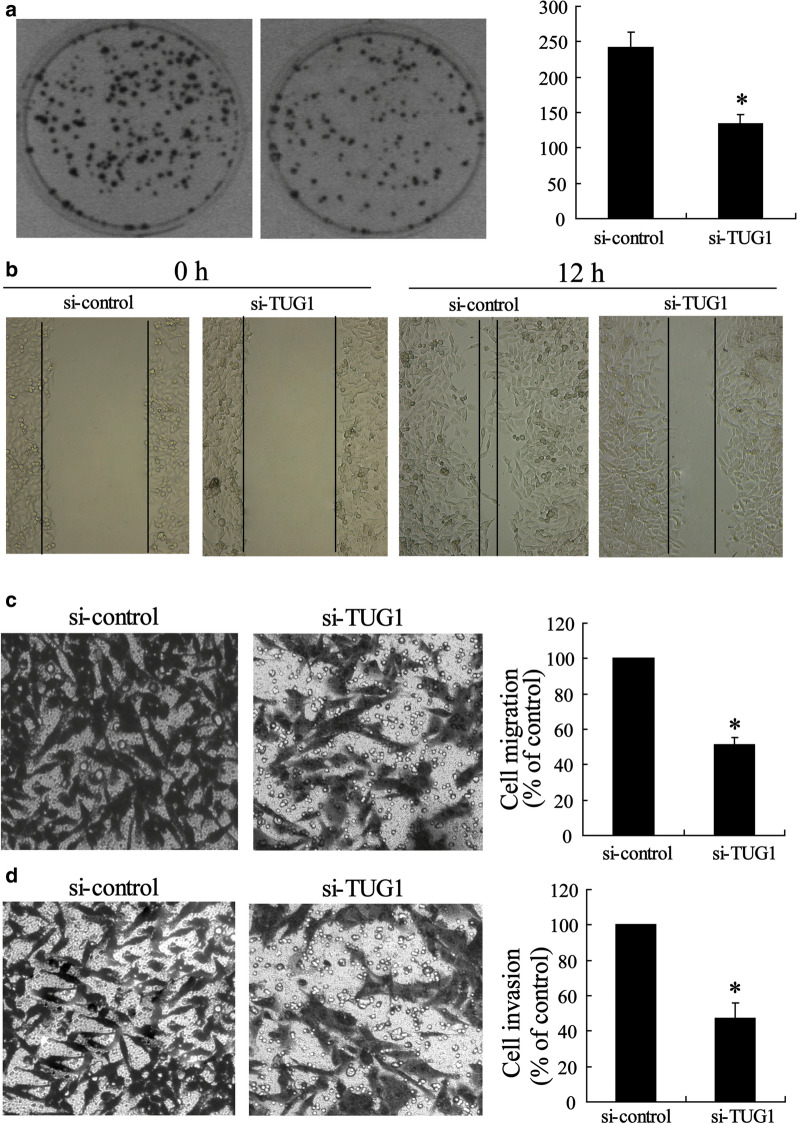# Correction to: The long non-coding RNA TUG1 indicates a poor prognosis for colorectal cancer and promotes metastasis by affecting epithelial-mesenchymal transition

**DOI:** 10.1186/s12967-020-02631-2

**Published:** 2020-12-22

**Authors:** Junfeng Sun, Chaohui Ding, Zhen Yang, Tao Liu, Xiefu Zhang, Chunlin Zhao, Jiaxiang Wang

**Affiliations:** 1 Gastrointestinal Surgery, The First Affiliated Hospital of Zhengzhou University, No.1 Jianshe East, Zhengzhou, 450052 China; 2 Pediatric Surgery, The First Affiliated Hospital of Zhengzhou University, No.1 Jianshe East, Zhengzhou, 450052 China

## Correction to: J Transl Med (2016) 14:42 https://doi.org/10.1186/s12967-016-0786-z

The authors found an error in the Figs. 3c–d and 4c–d of the original publication [1]. During the combination of the images of Figs. 3 and 4, the migration and invasion images were mixed up. The incorrect and correct figures are published in this correction article. The original article has been updated.

Correct Fig. 3

**Fig. 3:**
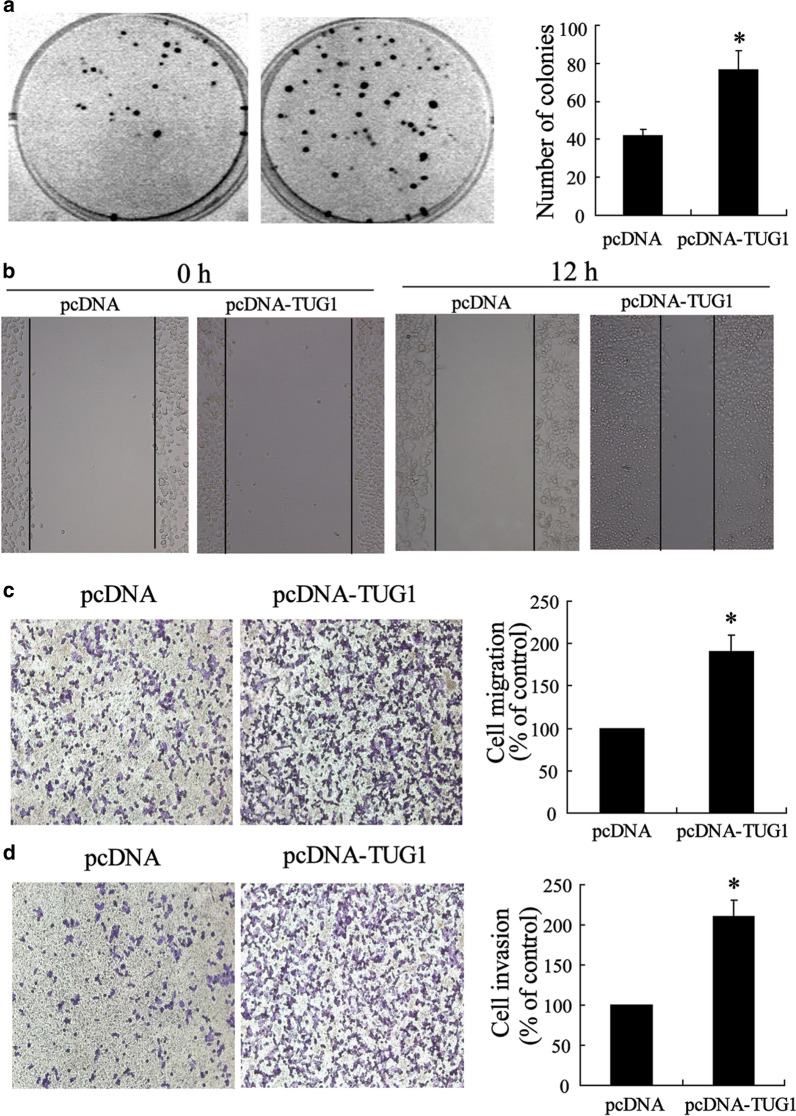
Enhanced metastasis of CRC cells with overexpressed TUG1. **a** Representative image and number statistics for colony formation in SW480^pcDNA^ and SW480^pcDNA-TUG1^ cells. **b** Wound-healing assay for motility of SW480^pcDNA^ and SW480^pcDNA-TUG1^ cells. Representative pictures of one field at the beginning (t = 0) (left panel) and at the end of the recording (t = 12 h) (right panel) in each condition. **c** Representative images of transwell migrated cells and d invaded cells in stably transfected SW480^pcDNA^ and SW480^pcDNA-TUG1^ cells and average number of migrated cells and invaded cells are shown in the right of (**c**) and (**d**). Values represent mean ± SD. *P < 0.05 compared with pcDNA

Incorrect Fig. 3
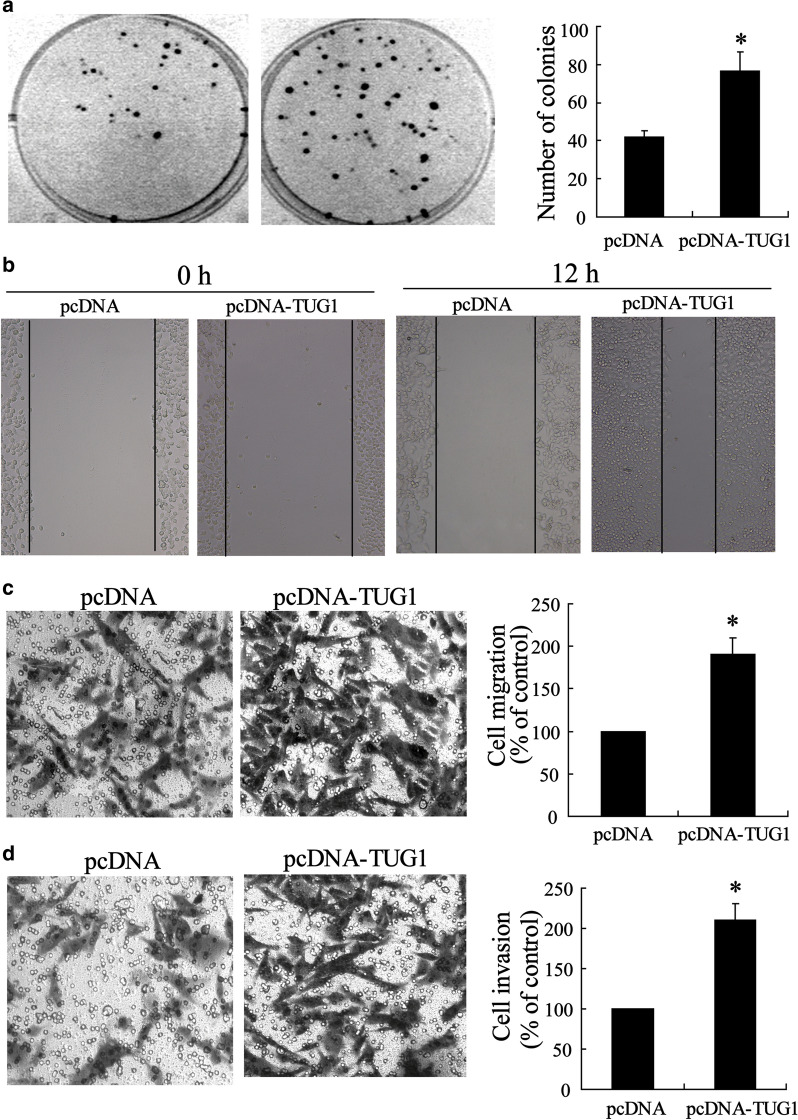


Correct Fig. 4

**Fig. 4:**
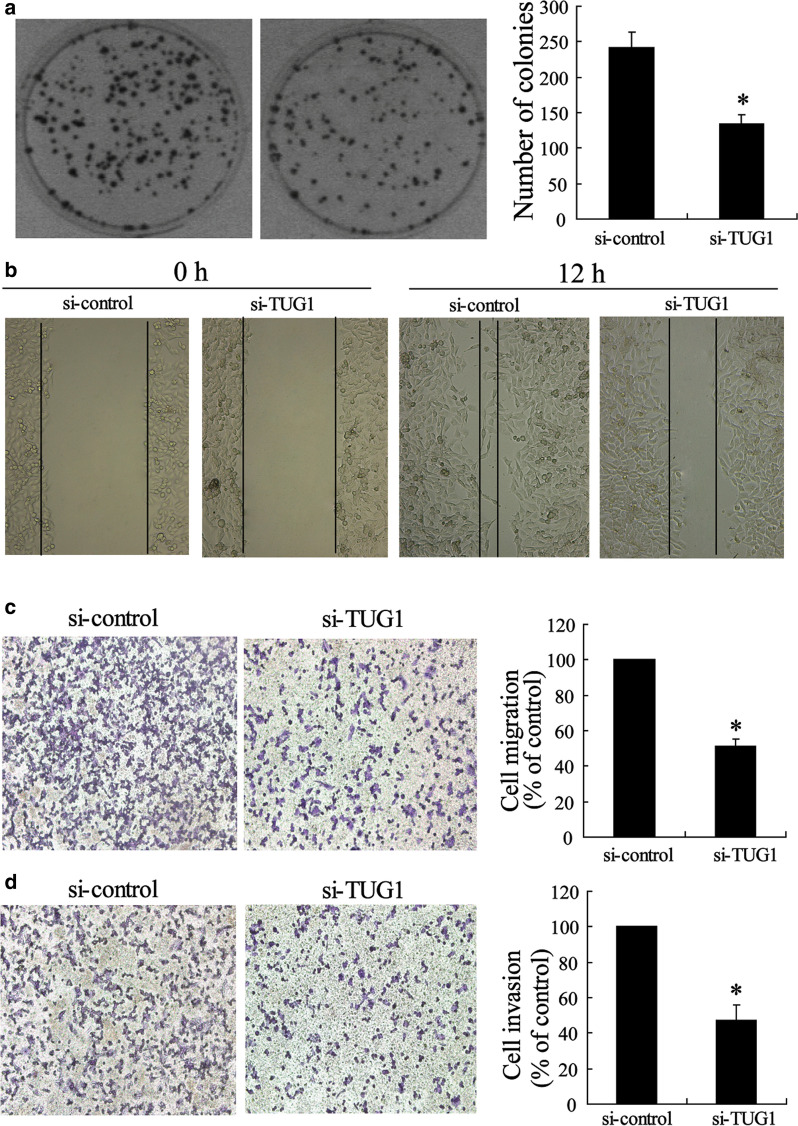
Silenced TUG1 inhibited metastasis of CRC cells. **a** Representative image and number statistics for colony formation in LOVO^si-control^ and LOVO^si-TUG1^ cells. **b** Wound-healing assay for motility of LOVO^si-control^ and LOVO^si-TUG1^ cells. Representative pictures of one field at the beginning (t = 0) (left panel) and at the end (t = 12 h) (right panel) of the recording in each condition are shown. **c** Representative images of transwell migrated cells, and d invaded cells in stably transfected LOVO^si-control^ and LOVO^si-TUG1^ cells and the average number of migrated cells and invaded cells are shown in the right of (**c**) and (**d**). Values represent mean ± SD. *P < 0.05 compared with si-control

Incorrect Fig. 4